# Association of Caudal-Type Homeobox 2 Immunohistochemical Expression With Pathological Stage, Tumor Grade, and Histological Subtype in Colorectal Malignancies: A Single-Center Observational Study

**DOI:** 10.7759/cureus.117620

**Published:** 2026-10-08

**Authors:** Archana Shrivastava, Varsha Rampuri, Paushali Nag

**Affiliations:** 1 Department of Pathology, Gandhi Medical College, Bhopal, IND

**Keywords:** cdx2, colorectal malignancy, immunohistochemistry (ihc), prognostic biomarker, ptnm stage, tumor grade

## Abstract

Background

Caudal-type homeobox 2 (CDX2) is a nuclear transcription factor that maintains intestinal epithelial differentiation. Reduced CDX2 expression has been associated with adverse pathological features and inferior outcomes in colorectal cancer, but its clinicopathological value depends on the scoring method and tumor context.

Methods

This single-center observational study evaluated 50 histopathologically confirmed colorectal malignancies. CDX2 immunohistochemistry was scored semi-quantitatively by multiplying nuclear staining proportion (0-4) by staining intensity (0-3), yielding an immunoreactivity score (IRS) of 0-12. Low/reduced expression was defined as IRS ≤4 and high/preserved expression as IRS ≥5. The patient-level master chart was used to summarize IRS and its associations with stage and grade; the histology comparison retained the original aggregated subtype counts. Exact tests used evaluable cases; pooled stage/grade analyses were exploratory, and subtype analysis was limited to major adenocarcinomas. The primary analytical endpoint was the association between CDX2 expression and pathological stage. The original pathology records for six cases recorded as pTis with N1/N2 disease were rechecked, and a conservative sensitivity analysis excluding these records was performed.

Results

CDX2 was evaluable in 48 of 50 cases; 29/48 (60.4%) showed low expression and 19/48 (39.6%) showed high expression. Median IRS was 2 (range 0-12). Among 43 cases with complete stage information and evaluable CDX2, low expression increased from 44.4% in stage 0, 30.0% in stage I, and 40.0% in stage II to 83.3% in stage III and 100% in the single stage IV case (Fisher-Freeman-Halton p=0.023). Advanced stage was associated with higher odds of low CDX2 expression (odds ratio (OR) 8.89, 95% confidence interval (CI) 2.01-39.22; p=0.004), although the wide confidence interval indicates imprecision. All 19 high-grade tumors showed low CDX2, compared with 9/27 (33.3%) low-grade tumors and 1/2 (50.0%) intermediate-grade tumors (p<0.001). Low CDX2 was also more frequent in mucinous adenocarcinoma (8/10, 80.0%) and signet-ring cell carcinoma (5/5, 100%) than in conventional adenocarcinoma (10/22, 45.5%; p=0.026). Pathology records confirmed that six entries combined primary lesions documented as pTis with separately submitted regional lymph nodes containing metastatic tumor. In a conservative sensitivity analysis excluding these six records, the early-versus-advanced-stage association persisted (low CDX2: 10/13 (76.9%) versus 9/24 (37.5%); OR 5.56, 95% CI 1.20-25.71; two-sided Fisher’s exact p=0.038), whereas the overall five-stage association is not statistically significant (exact p=0.182).

Conclusion

Reduced CDX2 expression tracked closely with advanced pathological stage, high tumor grade, and aggressive adenocarcinoma morphology. These findings support CDX2 as a practical adjunct marker of adverse tumor biology in colorectal malignancies. Because survival, recurrence, and molecular profiling were not evaluated, the present study supports clinicopathological prognostic association rather than independent survival prognostication. Because the cohort included different tumor classes and the detailed five-stage result was sensitive to the handling of six nonstandard pTis/node-positive records, the stage and grade findings should be considered exploratory.

## Introduction

Colorectal cancer remains a major cause of cancer-related morbidity and mortality and represents a biologically heterogeneous group of neoplasms. Its development reflects cumulative genetic and epigenetic alterations, while clinical behavior is strongly influenced by tumor stage, differentiation, histological subtype, lymph-node status, and metastatic spread [1,2]. Although pathological tumor, node, metastasis (pTNM) staging remains the backbone of prognostication, patients within the same stage can show substantially different biological behavior, creating interest in tissue biomarkers that complement conventional morphology.

Caudal-type homeobox 2 (CDX2) is an intestine-associated homeobox transcription factor expressed predominantly in the nuclei of intestinal epithelial cells. It contributes to epithelial identity, differentiation, and homeostasis, and experimental data support tumor-suppressive functions in the colorectum [3]. In colorectal neoplasia, reduced or absent CDX2 expression has repeatedly been associated with poor differentiation, mucinous or signet-ring morphology, advanced stage, and adverse molecular phenotypes [4-7]. Reviews and meta-analyses have further supported an association between preserved CDX2 expression and more favorable clinical outcomes, although the magnitude of prognostic effect varies across stage, molecular subtype, and scoring methodology [8,9].

The clinical interpretation of CDX2 is complicated by methodological heterogeneity. Studies have used complete absence, percentage-based thresholds, H-scores, and combined proportion-intensity systems to define low expression. A semi-quantitative immunoreactivity score (IRS) may capture biologically meaningful reductions that are not equivalent to complete immunonegativity. Recent clinicopathological studies using combined scoring have shown that low CDX2 can identify tumors with poor differentiation or advanced local stage even when complete loss is uncommon [10-12].

The present study was therefore designed to evaluate CDX2 immunohistochemical expression in colorectal malignancies and to determine whether reduced expression is associated with established adverse pathological features. For this manuscript, the primary analysis focused on pTNM stage, with tumor grade and major histological subtype as key secondary endpoints. The central hypothesis was that progressive pathological aggressiveness would be accompanied by reduced CDX2 expression. The stage and grade analyses were exploratory across the mixed colorectal-malignancy cohort, whereas the histological-subtype analysis was limited to conventional, mucinous, and signet-ring cell adenocarcinomas.

## Materials and methods

Study design, setting, and case selection

This was a single-center prospective observational study conducted in the Department of Pathology, Gandhi Medical College and Associated Hamidia Hospital, Bhopal, Madhya Pradesh, India. The study was conducted from May 2024 to October 2025 after obtaining approval from the Institutional Ethics Committee of Gandhi Medical College, Bhopal (IEC Protocol No. 183/IEC/2024; Reference No. 16295/MC/IEC/2024).

During the study period, consecutive histopathologically confirmed malignant lesions involving the colon and rectum received in the Department of Pathology were evaluated. Both biopsy and resection specimens were included. A total of 50 colorectal malignancy cases were analyzed. Cases from patients who had received chemotherapy or radiotherapy were excluded, in accordance with the study protocol.

Clinicopathological parameters including age, sex, tumor site, serum carcinoembryonic antigen (CEA) status when available, lymph-node status, depth of invasion, pathological tumor-node-metastasis (pTNM) classification, tumor grade, lymphovascular invasion, histological diagnosis, and CDX2 immunohistochemical expression were recorded in a structured patient-level dataset.

Histopathological evaluation

Tissues were fixed in 10% neutral-buffered formalin, processed, paraffin-embedded, sectioned, and stained with hematoxylin and eosin using standard histopathological methods [13]. Tumors were classified on the basis of the original histopathological diagnosis recorded in the master chart. pTNM categories and tumor grade were taken from the pathology dataset. For stage-focused reanalysis, cases with incomplete stage information (NA, TXNXMX, or MX preventing complete stage grouping) were excluded from the inferential stage table. Stage groups were summarized as stage 0, I, II, III, and IV according to the recorded pathological T, N, and M information. The American Joint Committee on Cancer (AJCC) Cancer Staging Manual, eighth edition, was used as the reference for TNM definitions [14]. Six records (cases 16, 24, 28, 31, 36, and 38) contained the nonstandard combination of pTis with N1/N2 and M0. The original primary-tumor and regional lymph-node pathology records for these cases were re-examined. The review confirmed that the primary colorectal lesions had been documented as pTis, whereas separately submitted regional lymph nodes contained metastatic tumor deposits and had been recorded as N1 or N2; therefore, the discordant entries were not attributable to a data-transcription or abstraction error. The separate submission of positive regional lymph nodes explains how the discordant entries arose but does not, by itself, make pTis with N1/N2 a conventional AJCC eighth-edition stage combination or exclude unrecognized invasion in the primary lesion. The recorded T and N categories were retained for transparent reporting and were grouped operationally with node-positive stage III disease in the exploratory analysis; however, they should not be interpreted as independently validated AJCC stage III assignments. A conservative sensitivity analysis excluding all six records was therefore performed.

Immunohistochemistry protocol

Immunohistochemistry (IHC) was performed on 3-4 μm sections mounted on positively charged slides. The laboratory protocol used a two-step horseradish peroxidase detection system (PathnSitu Biotechnologies Pvt Ltd, Hyderabad, India). Sections were deparaffinized in xylene and rehydrated through graded alcohols. Heat-induced epitope retrieval was performed using retrieval buffer in a pressure cooker. Endogenous peroxidase activity was blocked using hydrogen peroxide for five to 10 minutes.

A mouse monoclonal anti-CDX2 primary antibody was applied according to the validated laboratory protocol. After washing, target binder was applied followed by the horseradish peroxidase reagent. Diaminobenzidine was used as the chromogen, followed by hematoxylin counterstaining. Sections were dehydrated, cleared in xylene, and mounted with DPX. Appendix tissue served as the positive control. Only nuclear staining was considered specific for CDX2 [15].

CDX2 scoring

The proportion-intensity immunoreactivity score (IRS) framework was originally described by Remmele and Stegner [16]. The modified CDX2 proportion bands and cutoff used here are reported in the original colorectal cancer study by Xu et al. [17]. The proportion score was 0 for 0% positive tumor nuclei, 1 for 1-25%, 2 for 26-50%, 3 for 51-75%, and 4 for >75%. Staining intensity, compared with normal colonic epithelium, was scored as 0 (negative), 1 (weak), 2 (moderate), or 3 (strong). Multiplication of these two components produced an IRS ranging from 0 to 12. Consistent with the study protocol, IRS ≤4 was categorized as low/reduced expression and IRS ≥5 as high/preserved expression. The modified proportion bands and the CDX2-specific cutoff are attributed to Xu et al., rather than to the original breast-cancer IRS publication. The term 'low expression' is used throughout this manuscript rather than 'loss' because an IRS ≤4 can include weak or focal nuclear positivity and does not necessarily represent complete immunonegativity.

Outcomes and statistical analysis

The primary endpoint was the association between CDX2 expression category and pTNM stage. Key secondary endpoints were associations with tumor grade and major adenocarcinoma histology. The full 50-case cohort was retained for descriptive reporting. Each association analysis included only cases with evaluable CDX2 and the information required for that endpoint. Because the included tumor classes did not share uniform staging and grading systems, the pooled stage and grade analyses were considered exploratory. For the histology-focused inferential analysis, conventional adenocarcinoma, mucinous adenocarcinoma, and signet-ring cell carcinoma were analyzed because these groups had interpretable sample sizes; rarer mixed, metastatic, neuroendocrine, squamous, and non-epithelial tumors were described but were not included in that three-group exact test. The histological groups and their aggregated counts were retained from the original analysis: conventional adenocarcinoma (n=22), mucinous adenocarcinoma (n=10), and signet-ring cell carcinoma (n=5). Exact tests for this comparison were recalculated from these aggregated counts; case-level subtype allocation was not independently reassessed.

Categorical variables are presented as counts and percentages. IRS is also summarized by median, observed minimum-maximum range, and individual score frequencies overall and by recorded grade. Fisher-Freeman-Halton exact testing was used for multi-row contingency tables [18], and two-sided Fisher exact testing was used for 2x2 comparisons. For the revised calculations, all tables with the observed row and column totals were enumerated; the exact p-value is the sum of probabilities of tables no more likely than the observed table. The statistic reported for multi-row exact tests is Pobs, the conditional probability of the observed table, which is distinct from the summed p-value. No t, chi-square, or F-statistic is applicable to these exact tests. For the advanced-stage (III-IV) versus stage 0-II comparison and the pooled histological comparison, sample odds ratios (ORs) and log-Wald 95% confidence intervals (CIs) were calculated. A two-sided p-value <0.05 was considered statistically significant. The revised exact calculations used Python 3.12.14 (Python Software Foundation, Fredericksburg, VA) with integer combinatorial enumeration. Descriptive tables were not subjected to hypothesis testing.

Ethical approval

The study was approved by the Institutional Ethics Committee of Gandhi Medical College, Bhopal (IEC Protocol No. 183/IEC/2024; Reference No. 16295/MC/IEC/2024). Patient confidentiality was maintained, and no personal identifiers were included in the analytical dataset.

## Results

Cohort and CDX2 expression

The descriptive cohort comprised 50 cases, with a median age of 45 years (range 3-82 years); 32 patients (64.0%) were men and 18 (36.0%) were women. The original dataset contained a broad spectrum of colorectal malignancies, with conventional adenocarcinoma as the largest histological category. CDX2 IRS was evaluable in 48 cases, which constituted the CDX2-evaluable analytical cohort; the two cases with nonevaluable staining were excluded from all CDX2-based analyses (Table 1). Among the 48 evaluable cases, 29 (60.4%) showed low CDX2 expression (IRS ≤4), 19 (39.6%) showed high expression (IRS ≥5), and the median IRS was 2 (range 0-12).

**Table 1:** Demographic and clinicopathological characteristics of the study cohort CDX2, caudal-type homeobox 2; IRS, immunoreactivity score; pTNM, pathological tumor-node-metastasis. CDX2 expression was assessed using a semi-quantitative immunoreactivity score (IRS) ranging from 0 to 12. Low/reduced expression was defined as IRS ≤4, while high/preserved expression was defined as IRS ≥5. Stage groups were derived from available pathological T, N, and M information recorded in the study dataset. Complete recorded stage grouping was available for 44 cases; six cases had incomplete or indeterminate staging information and were categorized as pTNM stage incomplete/unclassifiable. CDX2 was evaluable in 48 cases, while the stage-CDX2 association analysis included 43 cases with both evaluable CDX2 staining and complete recorded stage information.

Characteristic	n	%
Total cases	50	100.0
Age, median (range), years	45 (3-82)	-
Male	32	64.0
Female	18	36.0
CDX2 low/reduced (IRS ≤4)	29	58.0
CDX2 high/preserved (IRS ≥5)	19	38.0
CDX2 not assessable	2	4.0
Derived pTNM stage 0	9	18.0
Derived pTNM stage I	11	22.0
Derived pTNM stage II	5	10.0
Derived pTNM stage III	18	36.0
Derived pTNM stage IV	1	2.0
pTNM stage incomplete/unclassifiable	6	12.0

Association of CDX2 with pathological stage

Among 43 cases with both evaluable CDX2 and complete stage grouping, the proportion of tumors with low CDX2 expression increased markedly in advanced disease. Low CDX2 was observed in 4/9 (44.4%) stage 0, 3/10 (30.0%) stage I, 2/5 (40.0%) stage II, 15/18 (83.3%) stage III, and the single stage IV tumor (100%). The overall association between stage group and CDX2 category was significant (Fisher-Freeman-Halton p=0.023) (Table 2).

**Table 2:** Association between pathological stage and CDX2 expression Analysis includes 43 cases with both evaluable CDX2 staining and complete recorded stage information. Of the 44 cases with complete stage grouping in Table 1, one case recorded as stage I MALT lymphoma lacked an evaluable CDX2 result and was excluded, accounting for the difference between 44 cases in Table 1 and 43 cases in this analysis. Percentages are calculated within stage groups. Test: Fisher-Freeman-Halton exact test; the merged statistic and p-value cells apply to the overall five-group comparison. Pobs is the conditional probability of the observed table, not the summed exact p-value. Six recorded but nonstandard pTis/node-positive entries were retained in the operational stage III group, as explained in the Methods; the effect of excluding these records is reported in the sensitivity analysis. Advanced stage (III-IV) versus stage 0-II: OR 8.89 (95% CI 2.01-39.22), two-sided Fisher’s exact p=0.004. The wide confidence interval indicates substantial imprecision due to the small number of cases.

pTNM stage	Total n	Low CDX2, n (%)	High CDX2, n (%)	Exact-test statistic	p-value
0	9	4 (44.4)	5 (55.6)	Pobs = 0.000203	0.023
I	10	3 (30.0)	7 (70.0)		
II	5	2 (40.0)	3 (60.0)		
III	18	15 (83.3)	3 (16.7)		
IV	1	1 (100.0)	0 (0.0)		

When stage 0-II tumors were grouped as early stage and stage III-IV tumors as advanced stage, low CDX2 was present in 9/24 (37.5%) early-stage tumors compared with 16/19 (84.2%) advanced-stage tumors. Advanced stage was associated with higher odds of low CDX2 expression (OR 8.89, 95% CI 2.01-39.22; Fisher’s exact p=0.004), although the wide confidence interval indicates substantial imprecision due to the small sample.

Association of CDX2 with tumor grade

Tumor grade showed the strongest association with CDX2 expression. All 19 high-grade tumors demonstrated low CDX2 (100%), whereas only 9/27 (33.3%) low-grade tumors had low expression. The two intermediate-grade tumors were evenly split between low and high CDX2. The three-level association was highly significant (Fisher-Freeman-Halton p<0.001) (Table 3). This pattern indicates a pronounced inverse relationship between the preservation of CDX2 and morphological dedifferentiation. Median IRS was 1 (range 0-2) in high-grade tumors, 4.5 (0-9) in intermediate-grade tumors, and 9 (0-12) in low-grade tumors.

**Table 3:** Association between tumor grade and CDX2 expression Analysis includes 48 tumors with evaluable CDX2; the GIST and MALT lymphoma without an IRS were excluded. Percentages are within-grade percentages. Test: Fisher-Freeman-Halton exact test. The merged statistic and p-value cells apply to the overall three-grade comparison. Pobs is the conditional probability of the observed table; the summed exact p-value is 0.00000217 (<0.001). Recorded grade categories were retained, including the two intermediate-grade neuroendocrine tumors.

Tumor grade	Total n	Low CDX2, n (%)	High CDX2, n (%)	Exact-test statistic	p-value
High grade	19	19 (100.0)	0 (0.0)	Pobs = 8.12 x 10^-7	0.00000217
Intermediate grade	2	1 (50.0)	1 (50.0)		
Low grade	27	9 (33.3)	18 (66.7)		

Association of CDX2 with histological subtype

The histology-focused analysis was restricted to the three major adenocarcinoma categories with adequate numbers. Conventional adenocarcinoma showed the highest preservation of CDX2, with high expression in 12/22 (54.5%) tumors. In contrast, low CDX2 was present in 8/10 (80.0%) mucinous adenocarcinomas and all 5/5 (100%) signet-ring cell carcinomas. The three-group association was significant (Fisher-Freeman-Halton p=0.026) (Table 4). Pooling mucinous and signet-ring carcinomas yielded low CDX2 in 13/15 (86.7%) compared with 10/22 (45.5%) conventional adenocarcinomas (OR 7.80, 95% CI 1.41-43.08; p=0.016).

**Table 4:** CDX2 expression in the major adenocarcinoma histological subtypes Test: Fisher-Freeman-Halton exact test of the three original histological groups. Percentages are within-subtype percentages. The merged statistic and p-value cells apply to the overall comparison. Pobs is the conditional probability of the observed table, not the summed exact p-value. The original aggregated subtype counts were retained and were not independently verified against the master dataset at the individual-case level. Rare mixed and non-adenocarcinoma histologies remain omitted from this focused comparison. Pooled mucinous and signet-ring carcinomas versus conventional adenocarcinoma: OR 7.80 (95% CI 1.41-43.08), two-sided Fisher exact p=0.016.

Histological subtype	Total n	Low CDX2, n (%)	High CDX2, n (%)	Exact-test statistic	p-value
Conventional adenocarcinoma	22	10 (45.5)	12 (54.5)	Pobs = 0.004765	0.026
Mucinous adenocarcinoma	10	8 (80.0)	2 (20.0)		
Signet-ring cell carcinoma	5	5 (100.0)	0 (0.0)		

The distribution of individual IRS values by recorded grade is shown in Table 5. Scores of 0, 1, 2, 8, 9, and 12 occurred in 8 (16.7%), 11 (22.9%), 10 (20.8%), 4 (8.3%), 11 (22.9%), and 4 (8.3%) of the 48 evaluable cases, respectively.

**Table 5:** IRS distribution and summary by recorded tumor grade IRS: immunoreactivity score. Score frequencies are n (%), calculated within each column. Scores 3, 4, and 6 were not observed; other integers between 0 and 12 are not attainable by this proportion-intensity product. All 48 evaluable cases are included. Descriptive data only; no hypothesis testing was performed.

IRS / summary	All evaluable (n=48)	Low grade (n=27)	Intermediate grade (n=2)	High grade (n=19)
0	8 (16.7)	1 (3.7)	1 (50.0)	6 (31.6)
1	11 (22.9)	2 (7.4)	0 (0.0)	9 (47.4)
2	10 (20.8)	6 (22.2)	0 (0.0)	4 (21.1)
8	4 (8.3)	4 (14.8)	0 (0.0)	0 (0.0)
9	11 (22.9)	10 (37.0)	1 (50.0)	0 (0.0)
12	4 (8.3)	4 (14.8)	0 (0.0)	0 (0.0)
Median (range)	2 (0-12)	9 (0-12)	4.5 (0-9)	1 (0-2)

Representative morphology and staining patterns demonstrate preserved nuclear CDX2 in a well-differentiated adenocarcinoma and reduced staining in a poorly differentiated adenocarcinoma (Figure 1). Representative mucinous and signet-ring morphology with reduced CDX2 staining is shown in Figure 2.

**Figure 1:**
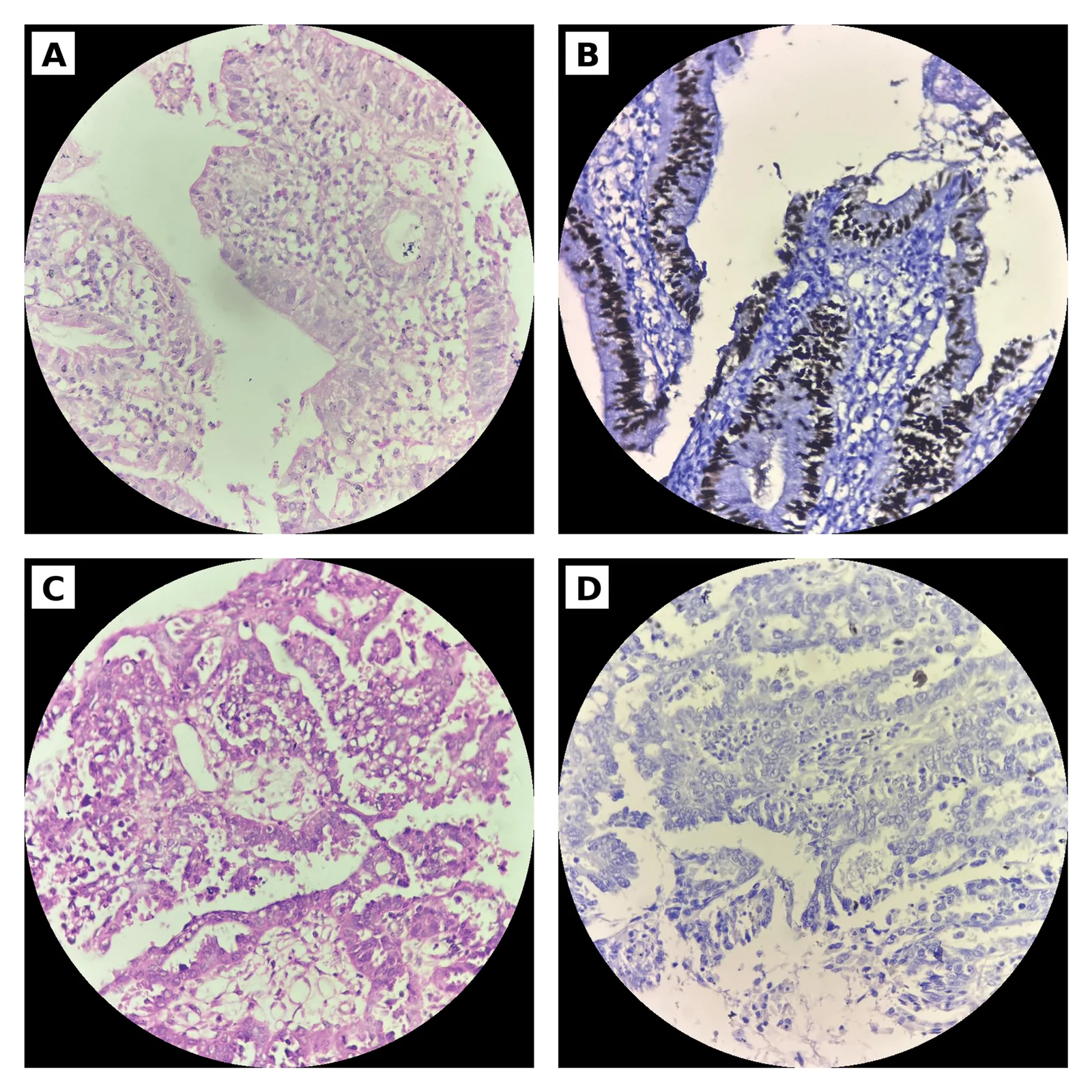
Representative histomorphology and CDX2 immunohistochemical expression in conventional colorectal adenocarcinoma (A) Well-differentiated conventional adenocarcinoma showing gland-forming tumor architecture on hematoxylin and eosin (H&E) staining. (B) Corresponding tumor showing preserved/high nuclear CDX2 immunohistochemical expression with strong nuclear staining. (C) Poorly differentiated adenocarcinoma demonstrating irregular glandular architecture, marked cytological atypia, and loss of differentiation on H&E staining. (D) Corresponding tumor showing reduced/low CDX2 immunohistochemical expression with absent or weak/focal nuclear staining. All images were captured at 400× total magnification (40× objective lens with 10× eyepiece lens). CDX2, caudal-type homeobox 2. Nuclear staining was considered specific for CDX2 expression. Representative photomicrographs are original images from the study specimens.

**Figure 2:**
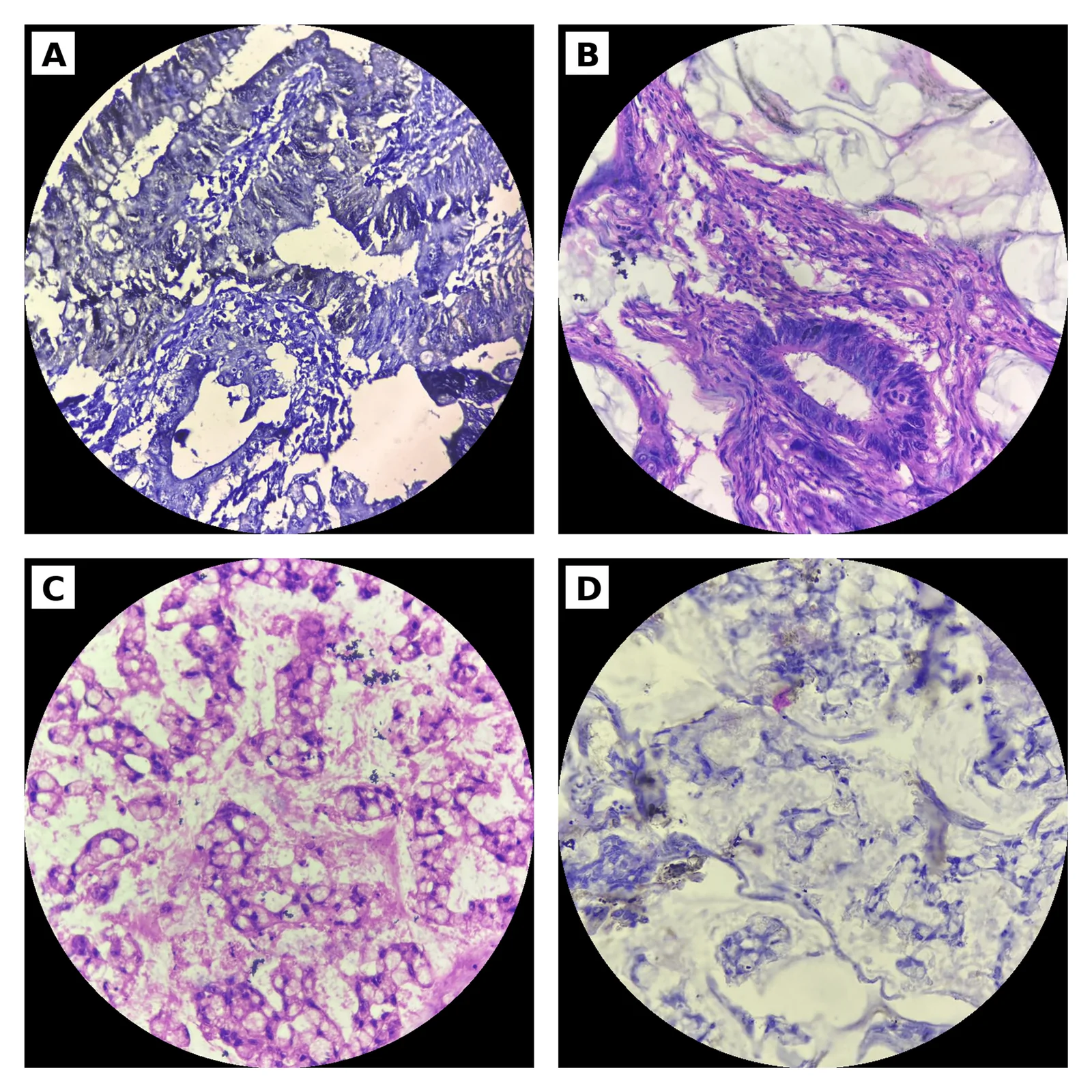
Representative aggressive adenocarcinoma variants and reduced CDX2 expression (A) Mucinous adenocarcinoma showing abundant extracellular mucin pools with malignant epithelial components on hematoxylin and eosin (H&E) staining. (B) Corresponding mucinous adenocarcinoma demonstrating reduced CDX2 immunohistochemical expression with weak/focal nuclear staining. (C) Signet-ring cell carcinoma showing scattered malignant signet-ring cells with intracytoplasmic mucin on H&E staining. (D) Corresponding signet-ring cell carcinoma showing markedly reduced/absent CDX2 immunohistochemical expression. All images were captured at 400× total magnification (40× objective lens with 10× eyepiece lens). CDX2, caudal-type homeobox 2. Nuclear staining was considered specific for CDX2 expression. Representative photomicrographs are original images from the study specimens.

## Discussion

The principal finding of this study is that reduced CDX2 expression is not randomly distributed across colorectal malignancies; rather, it clusters with established adverse pathological features. The most compelling associations were with pTNM stage and tumor grade. Low CDX2 was present in more than four-fifths of stage III-IV tumors compared with fewer than two-fifths of stage 0-II tumors, and every high-grade tumor in the evaluable cohort had a low IRS. This creates a coherent pathological gradient in which loss of intestinal differentiation, represented by declining CDX2, accompanies tumor dedifferentiation and disease advancement.

These observations are concordant with the biological role of CDX2. CDX2 is a lineage-defining transcription factor that helps maintain mature intestinal epithelial identity. Experimental work has shown tumor-suppressive functions for Cdx genes, and loss of CDX2 can facilitate dedifferentiation and a more proliferative, stem-like phenotype [3]. At the clinicopathological level, multiple cohorts have linked reduced CDX2 with high grade, advanced T or overall stage, and aggressive morphology [5-7,10-12]. The association therefore has mechanistic plausibility: a tumor that loses a key intestinal differentiation program is also more likely to display morphological and molecular features of aggressive progression.

The stage association in the present cohort is particularly notable. After excluding cases with incomplete stage grouping, 83.3% of stage III tumors and the single stage IV tumor had low CDX2, whereas high CDX2 predominated in stage I and II tumors. Although the point estimate suggested higher odds of low CDX2 expression in stage III-IV disease, the wide confidence interval indicates that the magnitude of this association is imprecisely estimated. Dalerba et al. demonstrated the clinical relevance of CDX2-negative tumors in stage II and III colon cancer, and subsequent studies have reported enrichment of low CDX2 in advanced disease [4,12,17]. Our findings extend that pattern in a single-center Indian pathology cohort using a semi-quantitative IRS rather than a strictly positive/negative definition.

Tumor grade produced an even sharper separation. All high-grade tumors showed low CDX2, compared with one-third of low-grade tumors. This closely parallels prior observations that CDX2 downregulation accompanies poor differentiation [5,6,11,12]. The complete separation seen in our high-grade subgroup should not, however, be interpreted as proof that CDX2 independently predicts outcome. Grade, stage, depth of invasion, histological subtype, and molecular phenotype are biologically interrelated, and the present sample size does not support a stable multivariable model capable of separating these effects.

Histological subtype also followed the expected biological pattern. Conventional adenocarcinoma retained CDX2 more often, whereas mucinous adenocarcinoma and signet-ring cell carcinoma showed progressively higher frequencies of low expression. Bae et al. and Baba et al. similarly reported associations between CDX2 reduction and mucin production, signet-ring morphology, poor differentiation, and aggressive pathological characteristics [5,6]. The particularly high frequency in signet-ring tumors in the present study is clinically plausible but is based on only five cases and should therefore be treated as a strong descriptive signal rather than a precise population estimate.

The overall frequency of low CDX2 in this cohort was 60.4% among evaluable tumors, substantially higher than the frequency of complete CDX2 absence reported in many large series. This difference is largely methodological and should not be conflated with complete immunonegativity. Our definition (IRS ≤4) intentionally captures weak and/or focal expression, whereas studies using a binary 'negative' definition generally report lower frequencies. For example, Bae et al. reported complete loss in 5.9% of 713 colorectal cancers, Baba et al. reported loss in 29% of 621 tumors using their study criteria, and a recent Vietnamese series using a combined IRS identified low expression in 11.2% [5,6,12]. Meta-analytic data also show wide heterogeneity according to scoring threshold, antibody methodology, tumor subtype, and cohort composition [9]. Accordingly, the present result should be interpreted as a high prevalence of reduced CDX2 by the study's IRS threshold, not as a 60% rate of complete CDX2 negativity.

At the molecular level, CDX2-low tumors frequently overlap with biological subgroups characterized by microsatellite instability, CpG island methylator phenotype, BRAF-pathway alterations, and right-sided or mucinous morphology, although the strength of these relationships varies among studies [6,7,19]. This is relevant to interpretation of CDX2 as a prognostic marker. CDX2 may function partly as an integrated phenotypic readout of differentiation and molecular context rather than as an isolated causal determinant of outcome. For that reason, CDX2 is most defensible as an adjunct to stage, grade, morphology, and molecular testing rather than a replacement for them [8,19].

The potential clinical utility is therefore in pathological risk stratification. In a tumor with otherwise concerning features, reduced CDX2 may reinforce the impression of aggressive biology and can be reported alongside established parameters. Conversely, preserved CDX2 supports maintained intestinal differentiation but should not be used to downstage or downgrade a tumor. Evidence regarding CDX2 as a predictive marker for adjuvant chemotherapy remains promising but inconsistent across tumor location and molecular subtype; treatment decisions should not be based on CDX2 alone [4,19].

Strengths and limitations

The strengths of this study include patient-level correlation of morphology with a reproducible proportion-intensity IRS, evaluation across multiple pathological stages and histological patterns, and availability of representative original histology/IHC images. Reanalysis from the master chart also allowed incomplete stage information and unevaluable CDX2 results to be handled explicitly rather than being merged with assessable cases.

Several limitations are important. The cohort was small and derived from a single center, with sparse numbers in several rare histologies and only one completely staged stage IV tumor. Biopsy and resection specimens were both included, creating non-uniform availability of lymph-node and depth-of-invasion information. The study did not evaluate overall survival, disease-free survival, recurrence, or treatment response, so it cannot establish CDX2 as an independent survival prognosticator. Molecular variables such as mismatch-repair/microsatellite-instability status, KRAS, BRAF, and CpG island methylator phenotype were not available. The primary antibody clone identifier was not documented in the archived protocol, which limits methodological reproducibility. Finally, multiple clinicopathological comparisons were explored in the thesis dataset; the present manuscript therefore emphasizes effect direction, exact p-values, and biological coherence rather than treating every nominally significant association as definitive. The stage III group retains six recorded but nonstandard pTis/node-positive combinations; these entries arose from separately submitted positive regional lymph nodes and were grouped operationally rather than independently validated as AJCC stage III. The five-stage association was sensitive to their conservative exclusion. Histological comparisons retain the original aggregated subtype allocation, which was not independently reassessed at case level. The cohort was pathologically heterogeneous, and its tumor classes did not share uniform staging or grading systems; pooled stage and grade analyses should therefore be regarded as exploratory. The wide confidence intervals around the stage and histology odds ratios indicate substantial imprecision. The archived materials also lacked complete antibody specifications and documentation of scorer number, blinding, interobserver assessment, disagreement resolution, and handling of heterogeneous staining.

## Conclusions

Reduced CDX2 expression was strongly associated with an adverse pathological phenotype in this cohort. Low CDX2 became substantially more frequent with advancing pTNM stage, was present in every high-grade tumor, and was enriched in mucinous and signet-ring cell carcinomas relative to conventional adenocarcinoma. These findings support CDX2 immunohistochemistry as a potential adjunct marker of tumor dedifferentiation and pathological aggressiveness; however, because the stage and grade analyses combined different tumor classes, these associations should be considered exploratory and should not be generalized uniformly to all colorectal malignancies.

The data should not be interpreted as demonstrating independent survival prognosis because longitudinal outcomes and multivariable molecular adjustment were not available. Larger, histologically homogeneous cohorts with standardized CDX2 scoring, molecular profiling, and overall/disease-free survival endpoints are required before CDX2 can be used as a stand-alone prognostic or treatment-predictive biomarker.
